# Superconductivity in a chiral nanotube

**DOI:** 10.1038/ncomms14465

**Published:** 2017-02-16

**Authors:** F. Qin, W. Shi, T. Ideue, M. Yoshida, A. Zak, R. Tenne, T. Kikitsu, D. Inoue, D. Hashizume, Y. Iwasa

**Affiliations:** 1 Quantum-Phase Electronics Center (QPEC) and Department of Applied Physics, The University of Tokyo, Tokyo 113-8656, Japan; 2 Materials Sciences Division, Lawrence Berkeley National Laboratory, Berkeley, California 94720, USA; 3 Faculty of Sciences, Holon Institute of Technology, 52 Golomb Street, PO Box 305, Holon 58102, Israel; 4 Department of Materials and Interfaces, Weizmann Institute of Science, Rehovot 76100, Israel; 5 RIKEN Center for Emergent Matter Science (CEMS), Wako, Saitama 351-0198, Japan

## Abstract

Chirality of materials are known to affect optical, magnetic and electric properties, causing a variety of nontrivial phenomena such as circular dichiroism for chiral molecules, magnetic Skyrmions in chiral magnets and nonreciprocal carrier transport in chiral conductors. On the other hand, effect of chirality on superconducting transport has not been known. Here we report the nonreciprocity of superconductivity—unambiguous evidence of superconductivity reflecting chiral structure in which the forward and backward supercurrent flows are not equivalent because of inversion symmetry breaking. Such superconductivity is realized via ionic gating in individual chiral nanotubes of tungsten disulfide. The nonreciprocal signal is significantly enhanced in the superconducting state, being associated with unprecedented quantum Little-Parks oscillations originating from the interference of supercurrent along the circumference of the nanotube. The present results indicate that the nonreciprocity is a viable approach toward the superconductors with chiral or noncentrosymmetric structures.

## Abstract (web summary)

Chirality affects many properties of materials, but how it affects superconductivity remains unclear. Here, Qin *et al*. report nonreciprocal supercurrent flows in individual nanotubes of WS_2_ via ionic gating, evidencing chiral superconducting transport.

Chirality of crystal or magnetic structures in solids was recently recognized as a powerful source of unique optical and electronic properties and novel functionalities. For instance, it is well known that polarization of light or spin of electron are sensitive to the chirality of lattice or magnetic structure^1^,^2^,^3^,^4^ and electric transport reflecting the chiral structure are also reported^5^,^6^,^7^. Among them, effects of chiral structures on superconductivity have not been investigated so far because of the lack of suitable materials. One of the interesting superconducting materials with chirality is carbon nanotube (NT)^8^,^9^. Chiral structures and their relations to the electronic properties in carbon NTs have been well studied by Raman scattering^10^,^11^, scanning tunnel microscope^12^ and even magneto-chiral transport^6^. However, since superconductivity in carbon NTs^13^,^14^,^15^,^16^ has been investigated only in the assembled form of single, double or multi-walled NTs, relations between superconducting transport and the chirality in individual tubes have remained elusive.

Tungsten disulfide (WS_2_) is a member of transition metal dichalcogenides (TMDs), which are now attracting significant attention as two dimensional (2D) materials beyond graphene with the potential application for electronics, photonics, spintronics, mechanics, as well as valleytronics^17^,^18^. Recent systematic studies clarified many TMDs including WS_2_, which are semiconductors without carrier doping, exhibit superconductivity under the ionic gating^19^,^20^. Importantly, TMD can form tubular structures with noncentrosymmetric chiral structures^21^,^22^,^23^,^24^,^25^,^26^. The semiconducting property of the WS_2_ NT was indeed demonstrated using the field effect transistor devices^24^. Superconductivity in such a noncentrosymmetric chiral cylinder, once it is realized, is a potential candidate for searching the exotic quantum phenomena and nontrivial Cooper pairing^27^,^28^. One of the manifestations of the chiral structure in the electronic transport is the unidirectional resistance. As shown in Fig. 1a,b, the two directions of current injection are not identical due to the chiral nature of the conducting substance when the magnetic field is applied parallel to the tube. Such nonreciprocity is highly anticipated to yield nontrivial quantum transport particularly in the superconducting states.

In this study, the transport properties of individual WS_2_ NT have been investigated by using the ionic liquid gating technique and resistance measurement on both first and second harmonic signals in alternative current (AC) mode. We have observed ambipolar transfer curve in electrostatic doping region and the emergence of superconductivity by electrochemical doping. The superconducting properties of individual WS_2_ NT have been further investigated, in which the observed anisotropy of the superconductivity and Little-Parks (LP) oscillation^29^ are consistent with tubular structure of WS_2_ NT. More importantly, we have experimentally discovered nonreciprocal superconducting transport via the second harmonic signal, being suggestive of chirality effect on superconductivity. Such nonreciprocal signal is largely enhanced in the superconducting state and affected by the magnetic flux quantum, showing periodic oscillations. The present study paves a route for studying the interplay between superconductivity and chirality or noncentrosymmetry.

## Results

### Sample characterization

**Figure 1:**
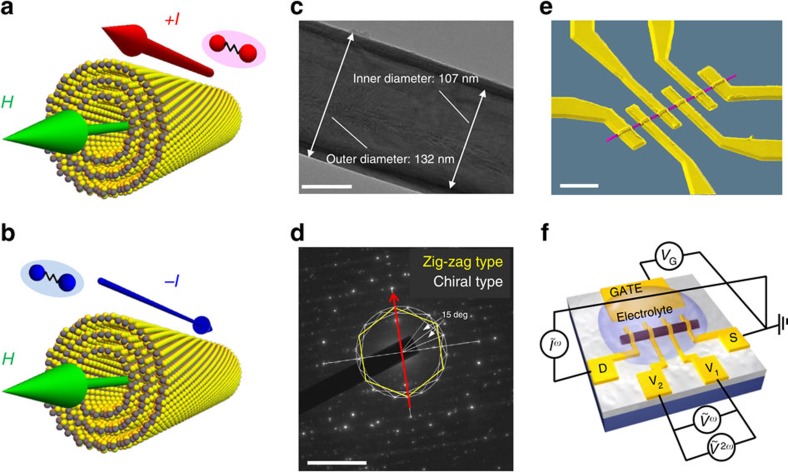
WS_2_ chiral NT and asymmetric superconducting transport in chiral NTs. (**a**,**b**) Illustrations of the unidirectional electric transport in superconducting chiral NT. Because of the broken inversion symmetry, asymmetric magnetoresistance is expected under magnetic field parallel to the tube axis, which can be probed via second harmonic signals of AC resistance. (**c**) TEM picture of a single WS_2_ NT. Contrast image shows the cylindrical structure with a diameter of about 100 nm. Scale bar, 50 nm. (**d**) Electron diffraction pattern of a single WS_2_ NT. Scale bar, 5 nm^−1^. The red arrow represents the direction of the tube axis. Tilted hexagonal diffraction pattern (white lines) confirms the existence of the chiral structure in addition to the zigzag type NT (yellow line) (see Supplementary Figs 5–9). (**e**) Coloured scanning electron microscope image (SEM) of a WS_2_ NT device. Scale bar, 2 μm. (**f**) Sketch of the electric double-layer transistor device. The electrolyte KClO_4_/polyethylene glycol is used as the gate medium. Both the first and second harmonic signals of the AC resistance have been measured by lock-in amplifiers (see ‘Methods' section).

WS_2_ NTs were synthesized following the literature^21^,^22^,^23^. Figure 1c shows a transmission electron microscope (TEM) image of a single WS_2_ NT (see Supplementary Fig. 1). The tube has a multi-walled structure, with the outer/inner diameters estimated as 132/107 nm, respectively, indicating that the layer number is ∼20. According to the literature^21^,^22^,^23^, the tube part has a 2*H*-polymorph-layered structure of WS_2_, where each tungsten atom is surrounded by six sulfur atoms in a trigonal biprism coordination (space group *P*63/*mmc*). The outer diameter distribution of tubes in the batch used for this measurement takes a broad maximum around 100 nm (See Supplementary Fig. 4). An electron diffraction pattern of a single WS_2_ NT is displayed in Fig. 1d. The red arrow and yellow hexagon represent the direction of tube axis and diffraction pattern from the zigzag type NT, respectively. The different walls of the tube can have different chirality. In addition to the contribution of the zigzag type NT, we can see the pair of tilted hexagonal pattern which confirms the co-existence of chiral structures in this NT. The TEM analysis indicates that the tubes used for the transport measurement are multi-walled WS_2_ NTs with chirality, having the outer diameter of nearly 100 nm (See Supplementary Fig. 4).

### Ionic liquid gating on WS_2_ NT and superconductivity

We fabricated an individual tube device as shown in Fig. 1e,f, and measured gate responses of the transport characteristics. Based on our previous research on the systematic study of superconductivity in TMDs^19^, we used KClO_4_/polyethylene glycol electrolyte as the gate medium to facilitate electrochemical intercalation of potassium ions into the layered structure of WS_2_.

**Figure 2:**
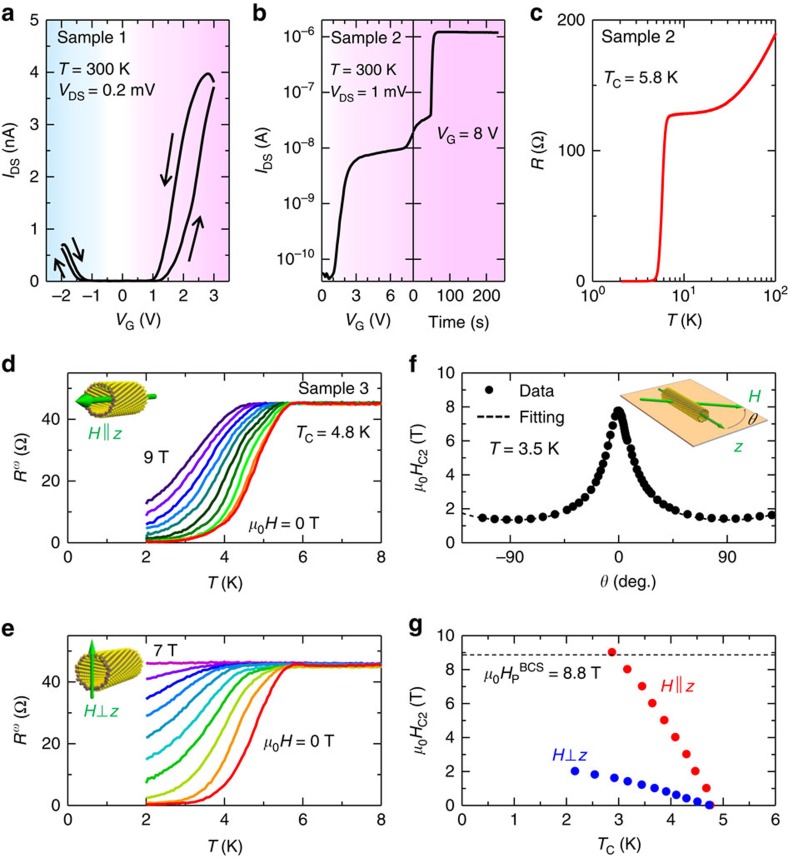
Ionic gating effect on WS_2_ NT and anisotropic superconducting behaviour. (**a**) Ambipolar transfer curve (*I*_DS_ versus *V*_G_) measured from sample 1. (**b**) *I*_DS_ as a function of *V*_G_ and waiting time measured from sample 2. First and second increase of *I*_DS_ observed at the electron-doped side can be attributed to the electrostatic and electrochemical doping, respectively^19^. (**c**) Superconducting transition after ionic gating with *V*_G_=8 V. *T*_c_ is 5.8 K, defined as the temperature corresponding to the half of the normal state resistance. (**d**,**e**) Temperature dependence of the resistance under magnetic field *H* parallel (**d**) and perpendicular (**e**) to the tube axis *z* measured from sample 3. (**f**) Angle dependence of the critical magnetic field *H*_c2_ measured at 3.5 K. Circles and dashed line represent the critical magnetic field obtained from the experiment and theoretical fitting by the anisotropic Ginzburg–Landau model. We define *θ*=0 degree when the magnetic field is parallel to the tube axis. (**g**) Temperature dependence of the perpendicular and parallel critical magnetic field *H*_c2_. Dashed line indicates the Pauli paramagnetic limit.

Figure 2a displays the source-drain current (*I*_DS_) of the individual WS_2_ NT device against the gate voltage (*V*_G_) between −2 and 3 V. The device nicely operates in an ambipolar mode, in a similar manner to the 2D devices, showing marked contrast with the unipolar response of WS_2_ NTs in the solid gated field effect transistor^24^. This indicates the strong gate coupling of the presently used ionic medium. The transistor operation is most likely in the electrostatic mode in this regime, considering the ambipolar behaviour is reversible and repeatable. When *V*_G_ was increased to 8 V at a constant rate of 50 mV s^−1^, we found a saturation of *I*_DS_, similarly to the case of 2D WS_2_ (ref. 19). When *V*_G_ was kept at 8 V for a couple of minutes, we encountered another dramatic increase of *I*_DS_ by more than two orders of magnitude as shown in Fig. 2b. This *I*_DS_ increase is presumably attributed to intercalation of K^+^ ions into WS_2_ NTs.

When we cooled down the device to 2 K keeping *V*_G_ at 8 V, superconductivity appeared at *T*_c_=5.8 K, defined as the temperature corresponding to the half of normal state resistance (Fig. 2c). In contrast to the K-intercalated 2D WS_2_ multilayer with *T*_c_ of 8.6 K (ref. 19), the superconducting transition here is shifted to lower temperature and considerably broadened, potentially due to the reduced dimensions or lack of commensurability between the different walls.

We then investigated the anisotropy of the observed superconductivity for sample 3 (*V*_G_=6 V). Figure 2d,e displays the temperature variation of the resistance under magnetic field *H* for *H*⊥*z* and *H*||*z*, respectively. Here *z* represents the tube axis direction. In the case of the *H*||*z*, the superconductivity is robust against the magnetic field and remains undefeated even under *μ*_0_*H*=9 T at *T*=2 K, while the superconducting phase rapidly disappears for the *H*⊥*z* configuration with increase of magnetic field. The anisotropic superconductivity was also confirmed by the angular dependence (Fig. 2f) and temperature dependence (Fig. 2g) of the upper critical magnetic field. In Fig. 2f, the estimated upper critical field at *T*=3.5 K are well fitted by the anisotropic Ginzburg–Landau model 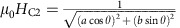 with fitting parameters *a*=0.13 T^−1^ and *b*=0.75 T^−1^. The temperature dependence of the critical magnetic field (Fig. 2g) cannot be explained either by the simple 2D or 3D models, implying that the system is of intermediate dimension. Here we should note that the upper critical field at *T*=0 K for the *H*||*z* configuration seemingly exceeds the Pauli paramagnetic limit 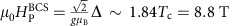 (Δ is the superconducting gap at *T*=0 K), being suggestive of a strong spin-orbit interaction and nontrivial Cooper pairing in the present system (See Supplementary Note 2).

### Little-Parks oscillations

**Figure 3:**
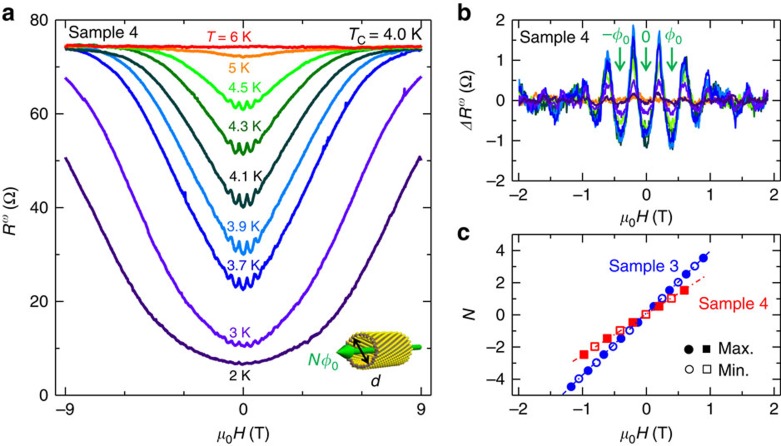
Little-Parks oscillations in the superconducting phase of WS_2_ NT. (**a**) Magnetoresistance measured from sample 4. *R*^*ω*^ denotes the first harmonic signal of the AC resistance (normal AC resistance measured by lock-in amplifier). LP oscillations appear when the magnetic field is applied along the tube axis (see inset cartoon), which indicates the interference of the superconducting current around the circumference. (**b**) Oscillating components calculated by subtracting the polynomial backgrounds from **a**. The oscillation shows a period of *φ*_0_ and enhanced magnitude around *T*_c_. (**c**) Index plot for the maximum and minimum peak positions of the LP oscillations. Open and closed circles (squares) correspond to the minimum and maximum positions for sample 3 (sample 4), respectively. We defined *N*=0 as the minimum peak position at zero magnetic field. From the slope of this plot, we can estimate the effective diameter *d* of the superconducting region, which is consistent with the values obtained from TEM observation.

**Figure 4:**
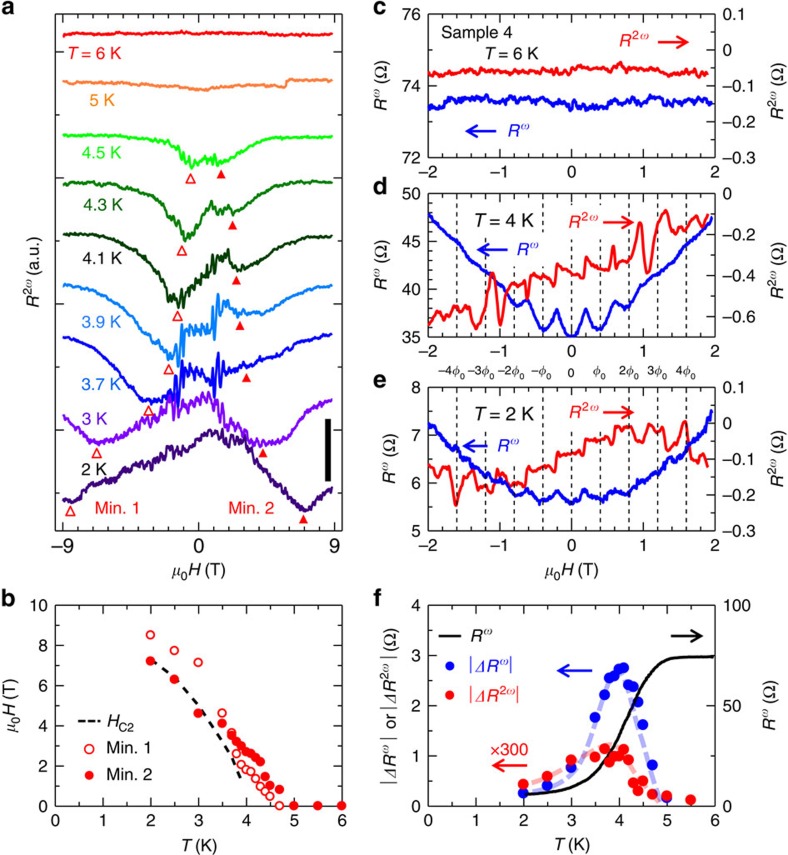
Nonreciprocal superconducting transport probed by second harmonic signals in AC magnetoresistance. (**a**) Magnetic field dependence of the second harmonic components in the AC resistance (*R*^2*ω*^) measured from sample 4. Scale bar, 0.5 Ω. Finite *R*^2*ω*^ signals reflecting the symmetry breaking in the chiral structure are observed during the superconducting transition. (**b**) Temperature dependence of upper critical magnetic field *H*_c2_ and characteristic minimum positions in *R*^*2ω*^. Each set of minimum positions gradually changes with temperature, showing similar behaviour as the critical magnetic field *H*_c2_. (**c**–**e**) Comparative plots of *R*^*ω*^ and *R*^*2ω*^ in low-magnetic field region at *T*=6, 4 and 2 K. Periodic oscillations were observed for both signals in the superconducting state (2 and 4 K), which disappears rapidly in the normal state (6 K). *R*^*2ω*^ shows the stepwise behaviour at low temperature (2 K). (**f**) Temperature variation of the resistance and magnitude of the quantum oscillations in *R*^*ω*^ and *R*^2*ω*^. For *R*^2*ω*^ oscillations, we estimate the magnitude of the jumps which are observed in low-field region. Both oscillating signals are enhanced around *T*_c_.

Figure 3a shows the AC magnetoresistance of sample 4 (*V*_G_=12 V) at various temperatures around *T*_c_ in *H*||*z* configuration. In addition to the robustness of the superconductivity discussed above, the magnetoresistance observed via the first harmonic signals in AC resistance (*R*^*ω*^) shows periodically oscillating behaviour in the low-magnetic field region. These oscillations during the superconducting transition known as LP effect^29^ originate from the interference of the superconducting current along the NT circumference and the resultant oscillations of *T*_c_ (refs 29, 30). During the application of a parallel magnetic field, the total flux piercing the NT should have a quantized value of *Nφ*_0_=*Nh*/2*e* and induce the oscillation of the free energy, manifested by the resistance oscillation with the period of *φ*_0_=*h*/2*e* as observed in Fig. 3a (*h* and *e* represent the Plank constant and charge of the electron, respectively, while *N* is an integer representing the number of flux quantum). Similar LP oscillations have been also observed in sample 3 (See Supplementary Fig. 11). We plotted the oscillating components at different temperatures in Fig. 3b after subtracting the polynomial background from Fig. 3a. The magnitude of the oscillating components reaches a maximum around *T*_c_ (see also Fig. 4f). From the periods of LP oscillations Δ(*μ*_0_*H*), we can estimate the effective diameter *d* of the superconducting NT to be 100 and 80 nm for sample 3 and 4, respectively, according to the relation 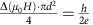 (Fig. 3c). This is consistent with the diameter distribution histogram of the same batch shown in Supplementary Fig. 4. These results provide firm evidence that superconductivity occurs in the tubular region in the present WS_2_ sample.

### Nonreciprocal superconducting transport in chiral WS_2_ NT

To clarify the characteristic properties due to the chiral structure, we have measured the second harmonic signals in the AC resistance (*R*^2*ω*^). In noncentrosymmetric systems, the cross term of magnetic field *H* and electric current *I* in the resistance is allowed on the basis of the symmetry argument, which indicates the difference between the forward and backward transports under magnetic field^5^,^6^,^7^. This term generates the nonlinear voltage response, which can be measured as the second harmonic components in the AC resistance (See Supplementary Note 3).

Especially in chiral systems, this nonreciprocal electric transport called magneto-chiral anisotropy has been reported in several materials with different chiral degrees of freedom^5^,^6^,^7^. Phenomenologically, magneto-chiral anisotropy can be expressed as


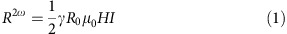


where both the magnetic field *H* and electric current *I* are parallel to the chiral axis and *γ* is the ratio of *R*^2*ω*^ to the normal resistance *R*_0_. So far, there has been no report of such a phenomenon in superconducting phase and it is of great interest whether excitation or quasiparticle in superconducting state without inversion symmetry generates such an unidirectional magnetoresistance.

Figure 4a shows the magnetic field dependence of second harmonic components *R*^2*ω*^ in the AC resistance measured in sample 4. Finite antisymmetric *R*^2*ω*^ signals were observed in the superconducting region, which unambiguously indicate the unidirectional electrical transport due to the chiral symmetry (Fig. 1a,b). In sharp contrast, *R*^2*ω*^ signals are negligibly small in the normal state (see Fig. 4c), indicating that magneto-chiral anisotropy signals are significantly enhanced in the superconducting phase due to the coherent nature of superconductivity.

The observed *R*^2*ω*^ signal has the two characteristic structures: that is, the broader antisymmetric components and the periodically oscillating terms in the low-magnetic field region. In the broader antisymmetric components, there are characteristic minima indicated by small triangles in Fig. 4a, which are dependent on temperature and enhanced at low temperature. The temperature dependence of the characteristic minima show similar behaviour as the critical magnetic field (Fig. 4b), strongly suggesting that the observed signals are related to the superconducting transition. (We show the details of temperature dependence of the antisymmetrized signals in the Supplementary Fig. 12 and discuss the possible co-existence of different chiral types in WS_2_ NTs in the Supplementary Note 3) The oscillating terms, on the other hand, show the periodicity of *φ*_0_ (Fig. 4c–e) and enhancement around *T*_c_ (Fig. 4f) similar to the LP oscillations (Fig. 3b), indicating that both have the same origin. Interestingly, this term shows the stepwise behaviour at low temperature (Fig. 4e, from *μ*_0_*H*=−1 T to 1 T) distinct from the conventional linear relation of the external magnetic field according to equation (1). This analysis indicates that the nonreciprocal supercurrent also has the interference nature and is affected by the flux quanta passing through the NT.

## Discussion

The observed second harmonic signals in AC resistance, together with LP oscillations in the first harmonic signals, are the direct manifestations of superconductivity in chiral NTs, however, the detailed mechanisms of the asymmetric electric transport and pairing symmetry (parity mixing) in the superconducting state needs to be further pursued. In light of this work, we expect various superconducting materials with broken inversion symmetry offer a similar transport, which provides a powerful approach for probing the exotic superconducting state in a variety of noncentrosymmetric systems.

## Methods

### Sample preparation

The WS_2_ NTs were synthesized following the literature^22^,^23^. The starting materials for the WS_2_ NT synthesis route were spherical tungsten oxide nanoparticles, which were sulfurized by solid-gas reaction with hydrogen and hydrogen sulfide at elevated temperatures (>800 °C). During this one-pot reaction, tungsten suboxide whiskers grow and are subsequently sulfurized into WS_2_ NTs of ∼100 nm in diameter and up to 20 micron in length. These two main steps of the reaction—oxide whiskers growth and sulfurization—occur under the same H_2_S/H_2_ gas flow regime and are not separated in space following each other in a self-controlled mechanism.

### Device fabrication

WS_2_ NTs were dispersed in isopropyl alcohol solvent by ultrasonication for 20 min. A droplet of the suspension was spin-coated on a Si/SiO_2_(3,000 Å) substrate, and immediately covered by polymethyl methacrylate. The isolated WS_2_ NTs were subsequently chosen by an optical microscope. The device pattern was designed via standard electron beam lithography techniques and developed by mixed solution of methyl isobutyl ketone and isopropyl alcohol with the ratio of methyl isobutyl ketone:isopropyl alcohol=1:3. After the deposition of Cr/Au (5 nm/90 nm), pads and gate electrode were covered by photoresist and Cr/SiO_2_ (5 nm/20 nm) was subsequently deposited to protect electrodes from the chemical reaction by ionic gating. Finally, we removed the redundant polymethyl methacrylate and gold by dipping the substrate into acetone for more than 1 h. KClO_4_/polyethylene glycol was selected as a gate medium^19^.

### Transport measurements

All the transport properties have been measured in a Quantum Design Physical Property Measurement System with a horizontal rotator probe under He-purged and high-vacuum environments. High-vacuum mode was used when the temperature was higher than 200 K, while the system was kept under He-purged condition below 200 K. Gate voltage was applied by a Keithley 2400 sourcemeter at 300 K with a sweeping rate of 50 mV s^−1^ under high-vacuum condition. Both the first and the second harmonic signals of the AC resistance have been measured by a lock-in amplifier (Stanford Research Systems Model SR830 DSP) with a frequency of 13 Hz. During the AC resistance measurements, the phase of the first (or second) harmonic signal was kept around 0 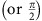, which is consistent with the theoretical expectation. All of the *R*^*ω*^ (or *R*^2*ω*^) signals discussed in the main text were obtained as x (or y)-component of the lock-in measurement.

### Data availability

All of the experimental data supporting this study are available from the corresponding author.

## Additional information

**How to cite this article:** Qin, F. *et al*. Superconductivity in a chiral nanotube. *Nat. Commun.* **8,** 14465 doi: 10.1038/ncomms14465 (2017).

**Publisher's note:** Springer Nature remains neutral with regard to jurisdictional claims in published maps and institutional affiliations.

## Supplementary Material

Supplementary Information Supplementary Figures 1-13, Supplementary Table 1, Supplementary Notes 1-3 and Supplementary References

## References

[b1] Rikken G. L. J. A. & Raupach E. Observation of magneto-chiral dichroism. Nature 390, 493–494 (1997).

[b2] Berova N., Nakanishi K. & Woody R. W. Circular Dichroism: Principles and Applications 2nd edn Wiley-VCH (2000).

[b3] Nagaosa N. & Tokura Y. Topological properties and dynamics of magnetic skyrmions. Nat. Nanotechnol. 8, 899–911 (2013). 24302027 10.1038/nnano.2013.243

[b4] Naaman R. & Waldeck D. H. Spintronics and chirality: spin selectivity in electron transport through chiral molecules. Annu. Rev. Phys. Chem. 66, 263–281 (2015). 25622190 10.1146/annurev-physchem-040214-121554

[b5] Rikken G. L. J. A., Fölling J. & Wyder P. Electrical magnetochiral anisotropy. Phys. Rev. Lett. 87, 236602 (2001). 11736466 10.1103/PhysRevLett.87.236602

[b6] Krstić V., Roth S., Burghard M., Kern K. & Rikken G. L. J. A. Magneto-chiral anisotropy in charge transport through sigle-walled carbon nanotubes. J. Chem. Phys. 117, 11315–11319 (2002).

[b7] Pop F., Auban-Senzier P., Canadell E., Rikken G. L. J. A. & Avarvari N. Electrical magnetochiral anisotropy in a bulk chiral molecular conductor. Nat. Commun. 5, 3757 (2014). 24796572 10.1038/ncomms4757

[b8] Iijima S. Helical microtubules of graphitic carbon. Nature 354, 56–58 (1991).

[b9] Harris P. J. F. Carbon Nanotube Science—Synthesis, Properties and Applications Cambridge Univ. Press (2009).

[b10] Rao A. M. . Diameter-selective Raman scattering from vibrational modes in carbon nanotubes. Science 275, 187–191 (1997). 8985007 10.1126/science.275.5297.187

[b11] Bandow S. . Effect of the growth temperature on the diameter distribution and chirality of single-wall carbon nanotubes. Phys. Rev. Lett. 80, 3779–3782 (1998).

[b12] Wildöer J. W. G., Venema L. C., Rinzler A. G., Smalley R. E. & Dekker C. Electronic structure of atomically resolved carbon nanotubes. Nature 391, 59–62 (1998).

[b13] Kociak M. . Superconductivity in ropes of single-walled carbon nanotubes. Phys. Rev. Lett. 86, 2416–2419 (2001). 11289943 10.1103/PhysRevLett.86.2416

[b14] Tang Z. K. . Superconductivity in 4 angstrom single-walled carbon nanotubes. Science 292, 2462–2465 (2001). 11431560 10.1126/science.1060470

[b15] Takesue I. . Superconductivity in entirely end-bonded multiwalled carbon nanotubes. Phys. Rev. Lett. 96, 057001 (2006). 16486971 10.1103/PhysRevLett.96.057001

[b16] Shi W. . Superconductivity in bundles of double-wall carbon nanotubes. Sci. Rep. 2, 625 (2012). 22953046 10.1038/srep00625 PMC3432458

[b17] Wang Q. H., Kalantar-Zadeh K., Kis A., Coleman J. N. & Strano M. S. Electronics and optoelectronics of two-dimensional transition metal dichalcogenides. Nat. Nanotechnol. 7, 669–712 (2012). 10.1038/nnano.2012.193 23132225

[b18] Xu X., Yao W., Xiao D. & Heinz T. F. Spin and pseudospins in layered transition metal dichalcogenides. Nat. Phys. 10, 343–350 (2014).

[b19] Shi W. . Superconductivity series in transition metal dichalcogenides by ionic gating. Sci. Rep. 5, 12534 (2015). 26235962 10.1038/srep12534 PMC4522664

[b20] Jo S., Costanzo D., Berger H. & Morpurgo A. F. Electrostatically induced superconductivity at the surface of WS_2_. Nano Lett. 15, 1197–1202 (2015). 25607653 10.1021/nl504314c

[b21] Tenne R., Margulis L., Genut M. & Hodes G. Polyhedral and cylindrical structures of tungsten disulphide. Nature 360, 444–446 (1992).

[b22] Rothschild A., Sloan J. & Tenne R. Growth of WS_2_ nanotubes phases. J. Am. Chem. Soc. 122, 5169–5179 (2000).

[b23] Zak A. . Scaling-up of the WS_2_ nanotubes synthesis. Fullerenes, Nanotubes and Carbon Nanostruct. 19, 18–26 (2010).

[b24] Levi R., Bitton O., Leitus G., Tenne R. & Joselevich E. Field-effect transistors based on WS_2_ nanotubes with high current-carrying capacity. Nano Lett. 13, 3736–3741 (2013). 23899194 10.1021/nl401675k

[b25] Panchakarla L. S. . Nanotubes from misfit layered compounds: a new family of materials with low dimensionality. J. Phys. Chem. Lett. 5, 3724–3736 (2014). 26278742 10.1021/jz5016845

[b26] Rao C. N. R. & Nath M. Inorganic nanotubes. Dalton Trans. 1, 1–24 (2003).

[b27] Bauer E. & Sigrist M. Non-Centrosymmetric Superconductors: Introduction and Overview Springer (2012).

[b28] Gor'kov L. P. & Rashba E. I. Superconducting 2D system with lifted spin degeneracy: mixed singlet-triplet state. Phys. Rev. Lett. 87, 037004 (2001). 11461584 10.1103/PhysRevLett.87.037004

[b29] Little W. A. & Parks R. D. Observation of quantum periodicity in the transition temperature of a superconducting cylinder. Phys. Rev. Lett. 9, 9–12 (1962).

[b30] Li L. J. . Controlling many-body states by the electric-field effect in a two-dimensional material. Nature 529, 185–189 (2016). 26700810 10.1038/nature16175

