# Supplementary material for: Superconductivity in a chiral nanotube
Source: Nat Commun. 2017 Feb 16;8:14465. doi: 10.1038/ncomms14465 (PMC5316891; doi:10.1038/ncomms14465)
Supplement: Supplementary Information — Supplementary Figures 1-13, Supplementary Table 1, Supplementary Notes 1-3 and Supplementary References [file ncomms14465-s1.pdf]

Supplementary Figures

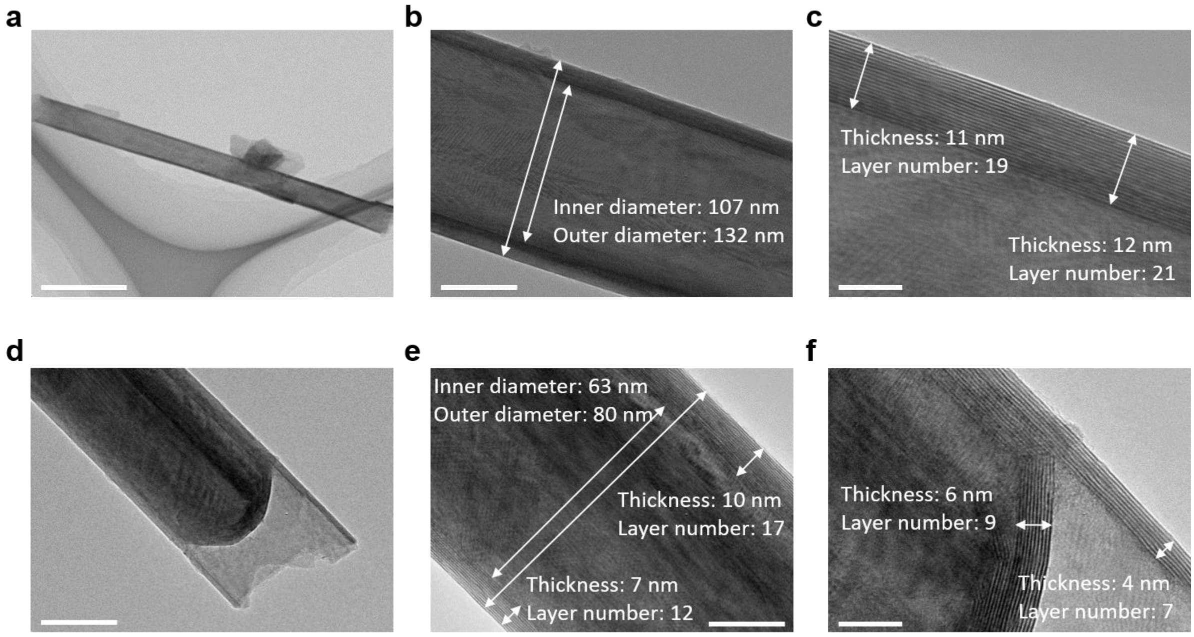

Supplementary Figure 1 | Transmission electron microscope (TEM) pictures of WS_2 Nanotubes (NTs).

(a) TEM image of an empty WS_2 NT. The scale bar is 500 nm. **(b)** Magnified view of Supplementary Fig. 1a. The scale bar is 50 nm. The inner and outer diameters of the NT are estimated as 107 nm and 132 nm, respectively. **(c)** Magnified view of Supplementary Fig. 1b. The scale bar is 10 nm. The number of layers varies from one position to the other along the tube, due to the stacking fault. **(d)** TEM image of another open ended WS_2 NT filled with a core material. The scale bar is 50 nm. **(e)** Magnified views of Supplementary Fig. 1d. The scale bar is 20 nm. The inner and outer diameters of the NT are estimated as 63 nm and 80 nm, respectively. The number of layers varies along the tube axis. No clear image of the core material was obtained. **(f)** Magnified views of Supplementary Fig. 1e. The scale bar is 10 nm. At the surface of the core material, a typical lamellar structure is observed, strongly suggestive of the existence of WS_2 also in the core material.

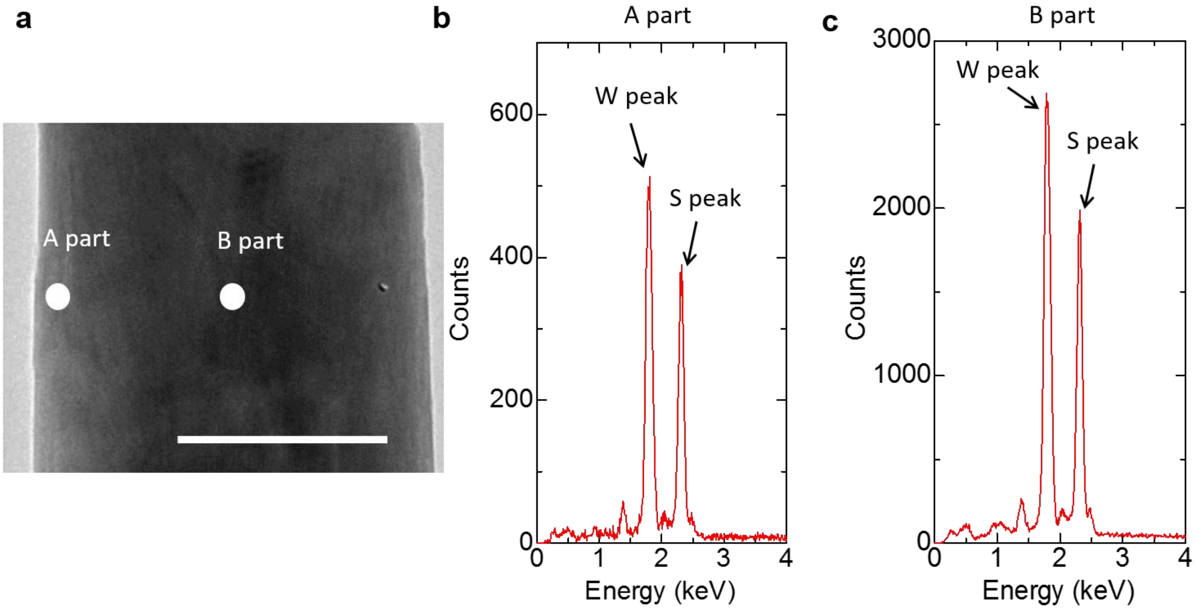

Supplementary Figure 2 | WS_2 NT with core-shell structure and energy dispersive X-ray spectroscopy (EDS) analysis.

(a) TEM image of a WS_2 NT filled with a core material (the scale bar, 100 nm). **(b)** EDS spectra of A part in Supplementary Fig. 2a. **(c)** EDS spectra of B part in Supplementary Fig. 2a. Both parts basically show only tungsten and sulfur peaks, indicating a complete sulfurization of the seed oxide WO_x.

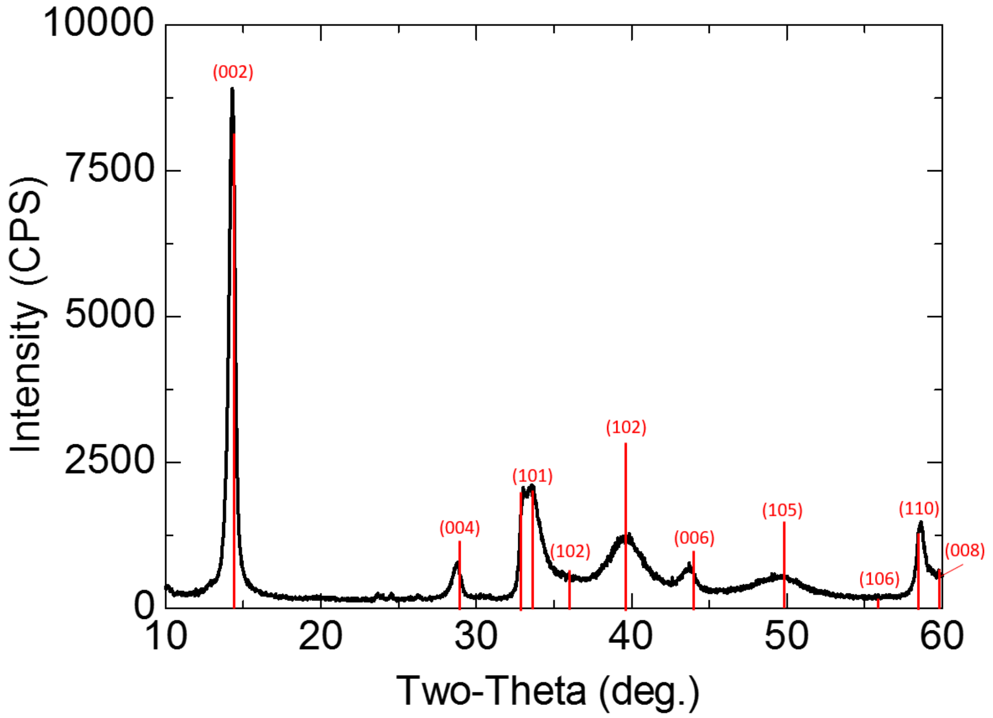

Supplementary Figure 3 | X-ray diffractogram of bulk sample of WS_2 NT, recorded with a Cu-Kα source.

Most of the dominating peaks are indexed by the 2*H* WS_2 structure (red lines), indicating that both core and shell are basically WS_2. The downwards shift of the (00*l*) peaks (compared to the bulk 2*H* phase) represents a small (*ca*. 2%) interlayer expansion which is typical for such nanotubular structures.

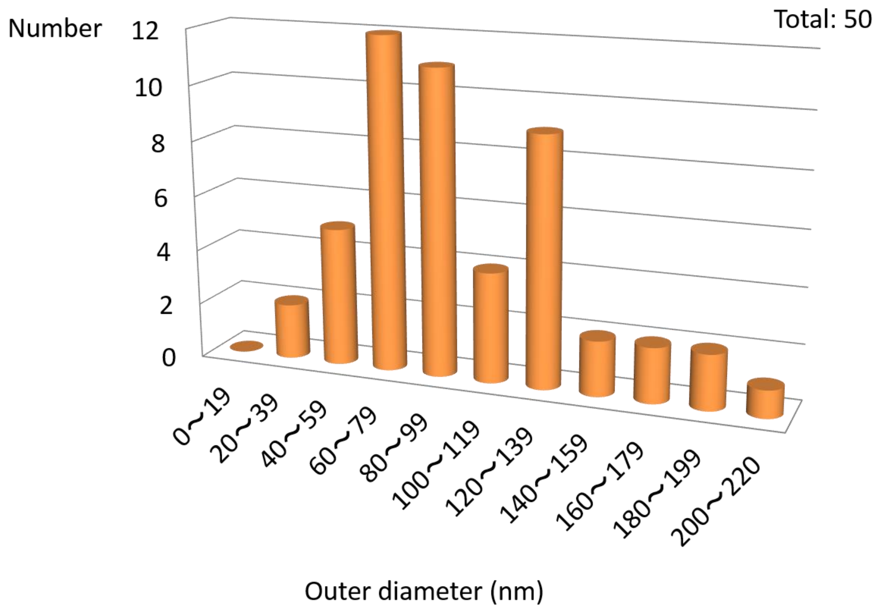

Supplementary Figure 4 | The diameter distribution of WS_2 NTs.

The total number of samples is 50 and median of the diameter value is about 100 nm.

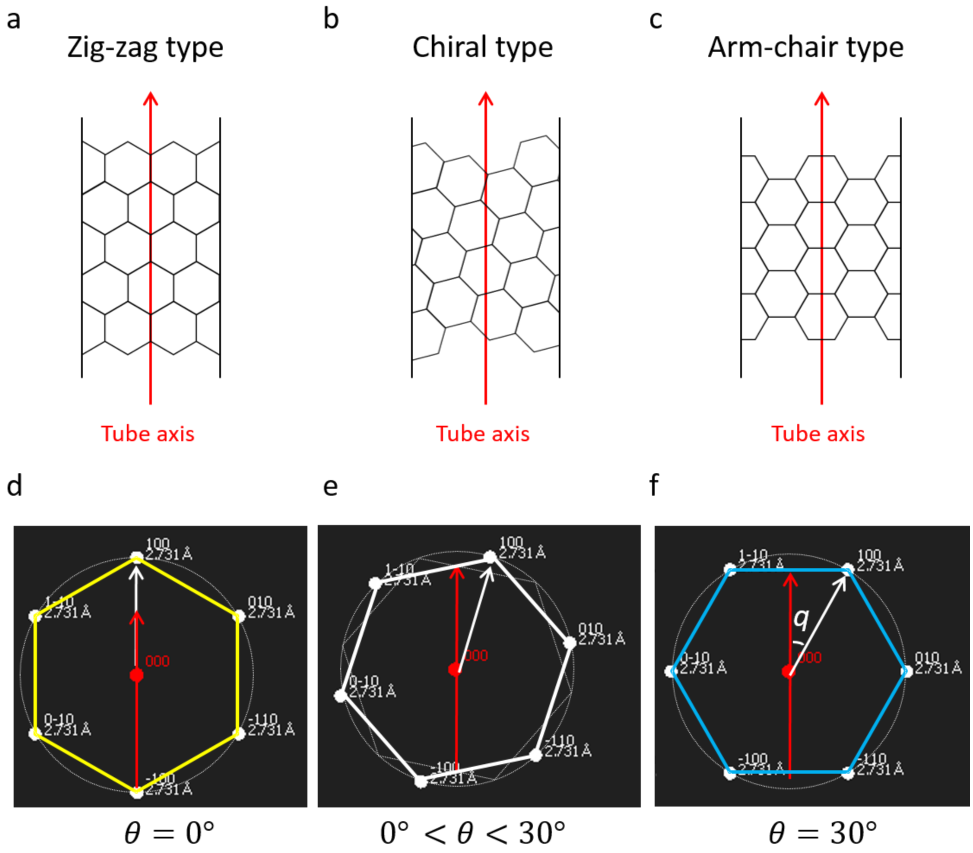

Supplementary Figure 5 | Expected electron diffraction patterns.

(a) – **(c)** Illustration of the three typical NT structures (**(a)** zig-zag type, **(b)** chiral type, and **(c)** arm-chair type). Red arrow represents the tube axis direction. **(d)** – **(f)** Expected diffraction pattern for each structure (**(d)** zig-zag type, **(e)** chiral type, and **(f)** arm-chair type). **(d)** For the zig-zag NT, the diffraction pattern is made up of six points with zero chiral angle. **(e)** In case of chiral NT, the diffraction pattern is composed of two sets of six points, each of which originates from the top and bottom layer of the NT. **(f)** For the arm-chair NT, the diffraction pattern is also made up of six points with chiral angle of $30°$.

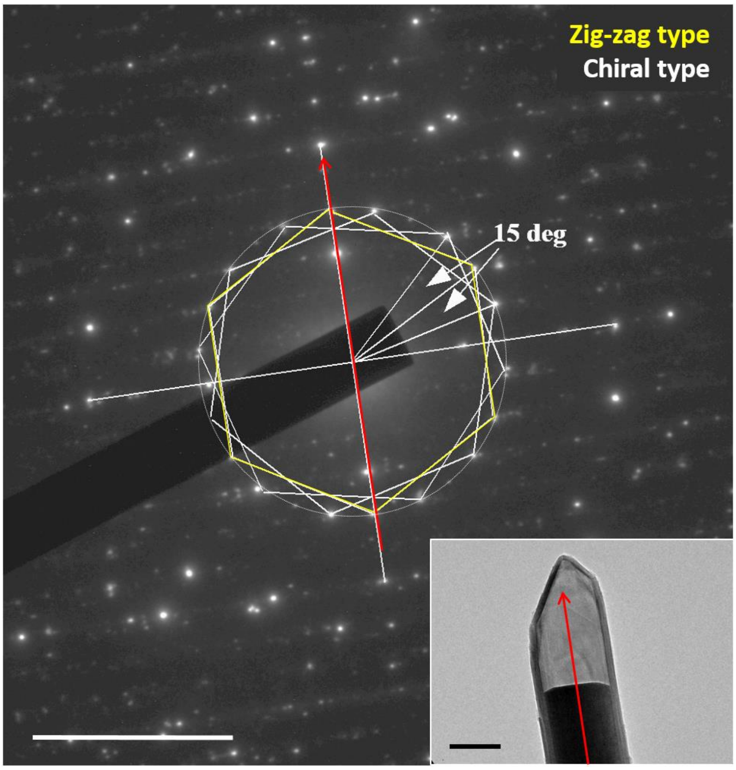

Supplementary Figure 6 | Electron diffraction pattern of tube A.

There are zig-zag type and chiral type walls coexisting in the multi-wall tubular structure according to the electron diffraction pattern of sample A. The scale bar is 5 nm^{-1}. The inset shows the TEM image of the apex of this tube. The scale bar is 50 nm.

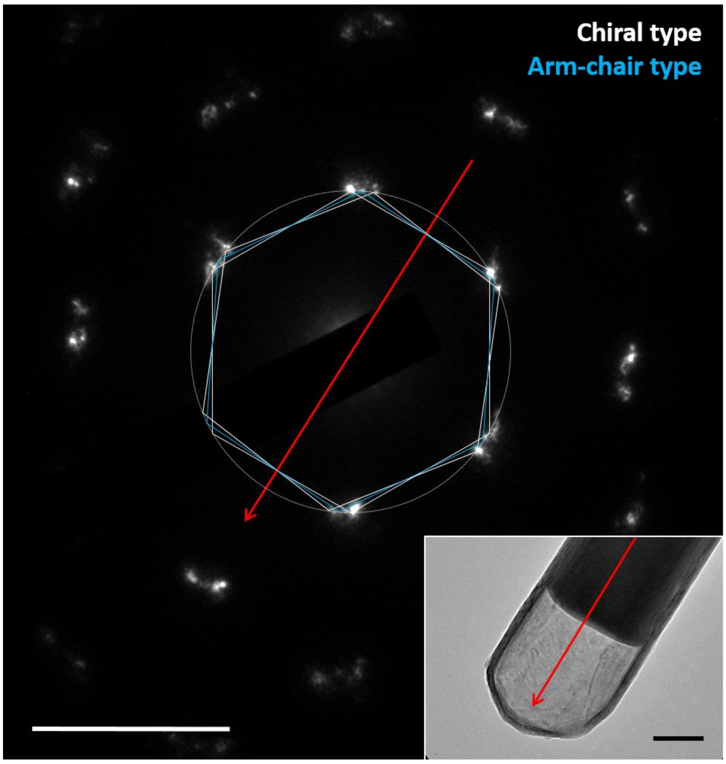

Supplementary Figure 7 | Electron diffraction pattern of WS_2 tube B.

There are arm-chair type and chiral type walls coexisting in the multi-wall structure from the electron diffraction pattern of sample B. The scale bars are 5 nm^{-1} for the diffraction pattern and 50 nm for the inset, respectively.

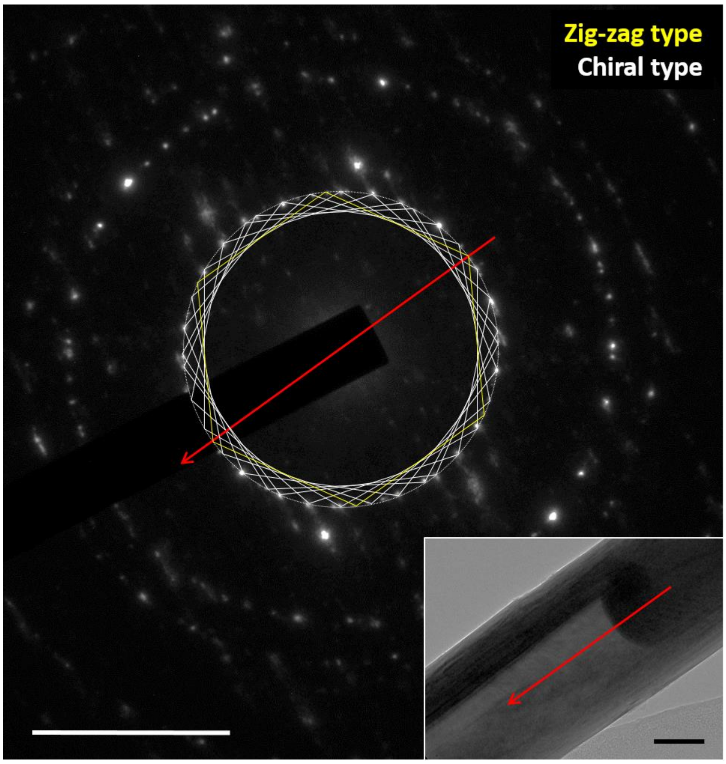

Supplementary Figure 8 | Electron diffraction pattern of tube C.

The zig-zag type and multi-chiral type walls coexisting in the multi-wall tubular structure concluded from the electron diffraction pattern of sample C. The scale bars are 5 nm^{-1} for the diffraction pattern and 50 nm for the inset.

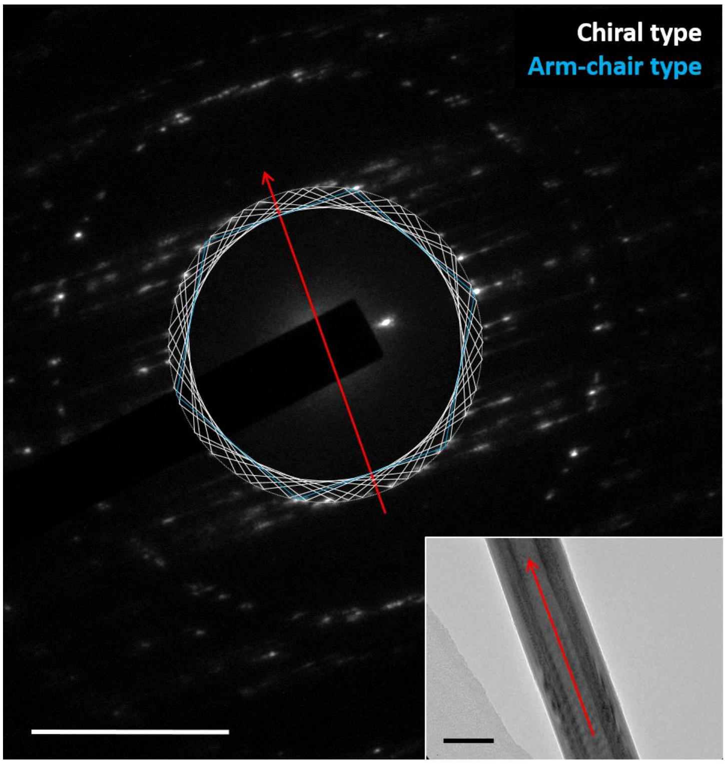

Supplementary Figure 9 | Electron diffraction pattern of tube D.

Electron diffraction of sample D shows that this NT consists of arm-chair type and multi-chiral type walls. The scale bars are 5 nm^{-1} for the diffraction pattern and 50 nm for the inset.

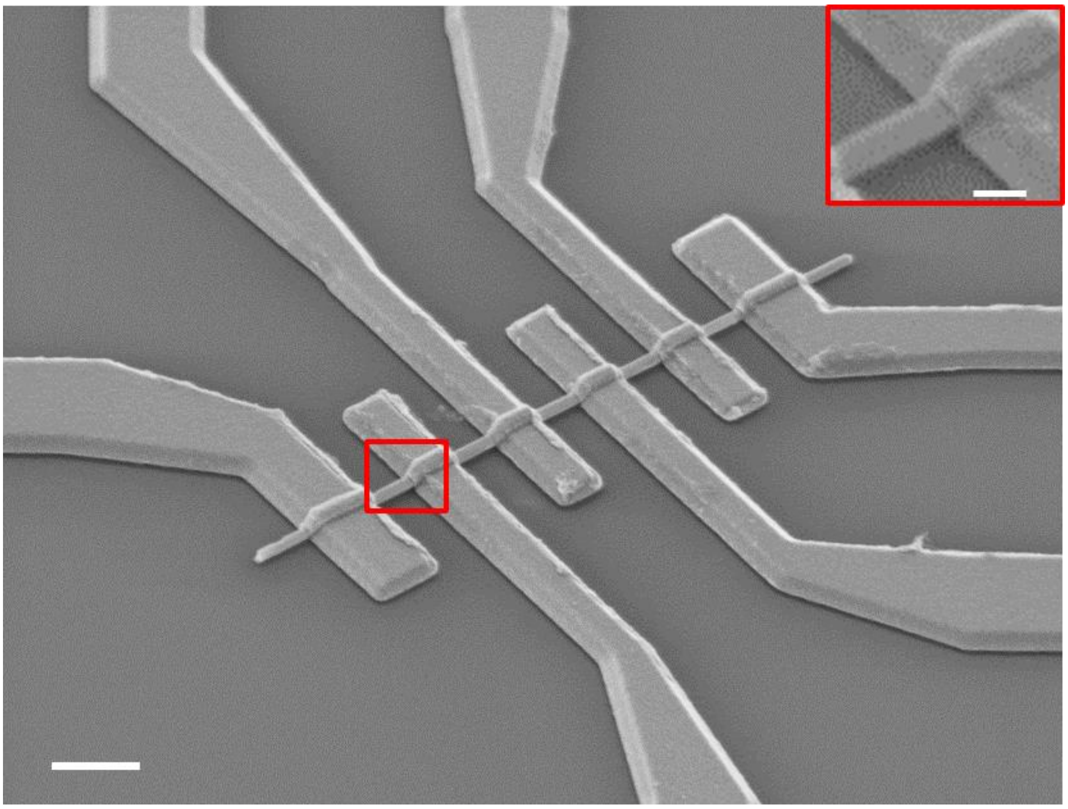

Supplementary Figure 10 | Scanning Electron Microscope (SEM) image of a WS_2 NT device.

Inset shows the magnified view around the boundary between WS_2 NT and gold electrode. The scale bars are 1 μm for the device picture and 200 nm for the inset.

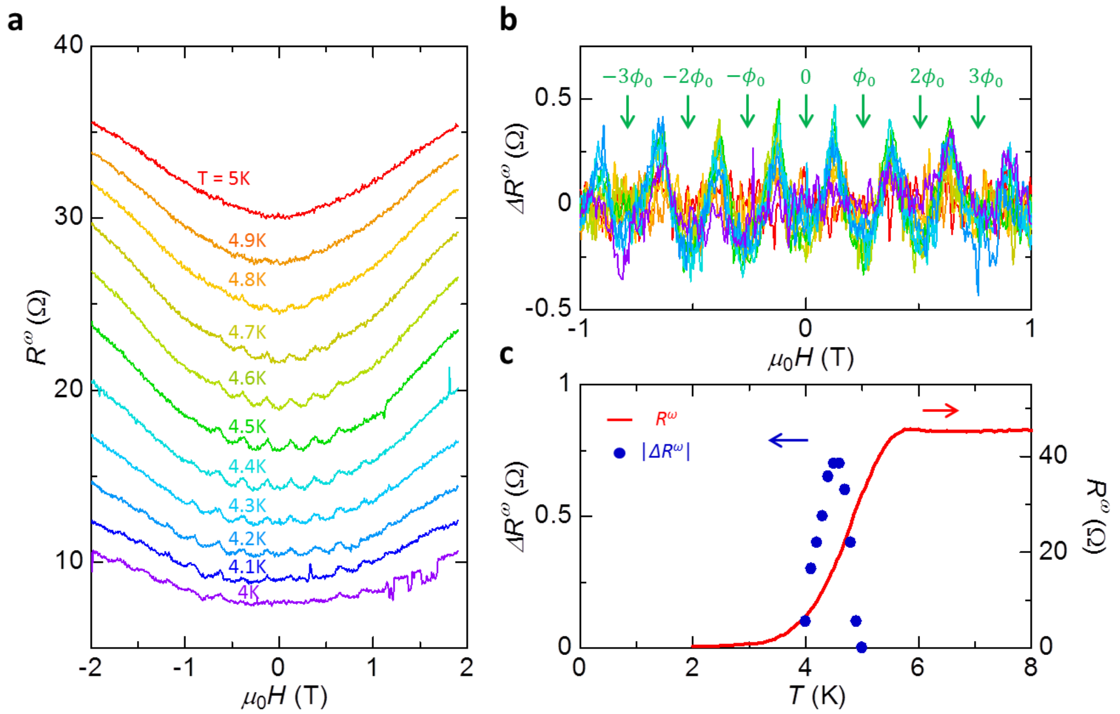

Supplementary Figure 11 | Little-Parks effect in sample 3.

LP oscillations are also observed in sample 3. **(a)** Raw data of the magnetoresistance in the low field region. **(b)** The oscillating part calculated by subtracting the polynomial backgrounds from Supplementary Fig. 11(a). **(c)** Temperature variation of the resistance and magnitude of LP oscillations. The period of oscillations is estimated as 100nm which is consistent with the AFM result of 125nm. The maximum magnitude of the oscillating components (Supplementary Fig. 11b) is around T_c (Supplementary Fig. 11c).

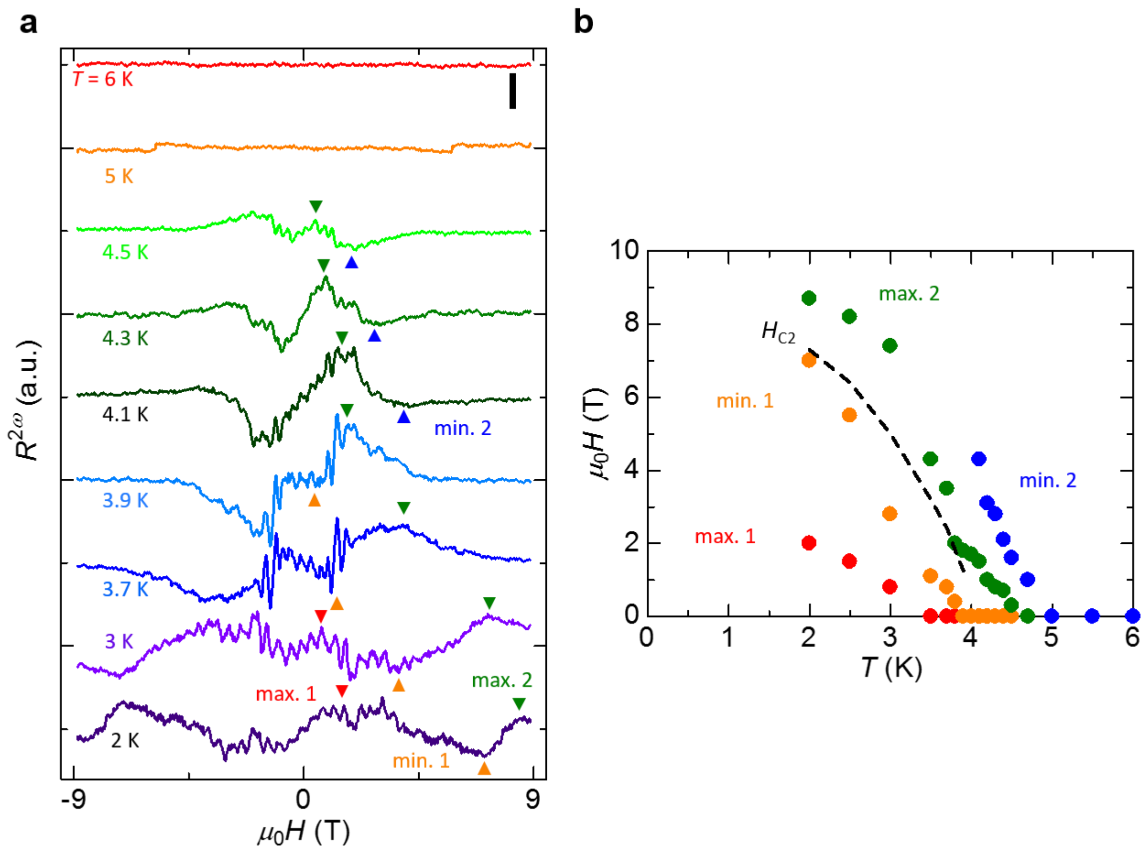

Supplementary Figure 12 | Antisymmetrized components of the second harmonic signal in alternative current (AC) mode of sample 4.

(a) Magnetic field dependence of the antisymmetrized second harmonic component in the AC resistance. The scale bar is 0.2 Ω. The finite $R^{2\omega}$ signals originating from the lattice symmetry breaking are observed during the superconducting transition. **(b)** Critical magnetic field and characteristic magnetic field positions of the antisymmetric components observed in the broader field range as a function of temperature. Maximum or minimum positions depend on temperature, each of which potentially corresponds to the nonreciprocal signals from superconductivity with different chirality, T_c and H_{c2}.

a b

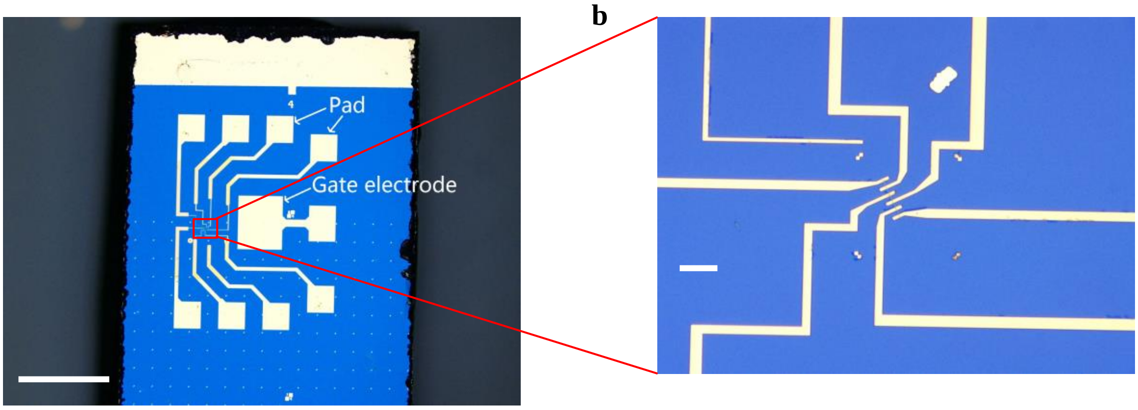

Supplementary Figure 13 | Photographs of sample 4

We show the photographs of sample 4 below. As illustrated in Fig. 1f in main text, the gate electrode is positioned near the nanotube. **(a)** Photograph of device of sample 4. The scale bar is 0.5 mm. **(b)** Magnified view of Supplementary Fig. 13a. The scale bar is 10 μm.

Supplementary Table

Element	Peak position (keV)	A part: Content (%)	B part: Content (%)
W	1.774	35.1	36.3
S	2.307	64.9	63.7

Supplementary Table 1| Quantitative analysis of A part and B part in Supplementary Figure 2a.

For both parts, the composition ratio is almost W:S = 1:2.

Supplementary Notes

Supplementary Note 1: Characterization of WS_2 Nanotube (NT) device

The structure of the WS_2 NTs was determined by transmission electron microscope (TEM) and related techniques, including electron diffraction (ED) and energy dispersive X-ray spectroscopy (EDS). Supplementary Fig. 1 shows details of two typical NTs with a similar outer diameter but with different core characteristics. TEM images were taken by JEM-2100F (JEOL Ltd.) with accelerating voltage of 200 kV.

Supplementary Fig. 1a, 1b and 1c show empty multi-walled WS_2 NTs. More than 90 % of the WS_2 NTs we observed had a core-shell structure as shown in Supplementary Fig. 1d, 1e and 1f. In both types of NTs, the shell showed a very nice lamellar structures with the interlayer separation of approximately 0.7 nm, and this value is close to that in 2*H* WS_2. In contrast to the well-ordered shell part, the core part did not show any well-ordered patterns, as seen in Supplementary Fig. 1e. However, we occasionally saw lamella structures on the surface of the core materials as in Supplementary Fig. 1f. This might suggest that the core is materials also composed of WS_2.

According to the EDS analysis (Supplementary Fig. 2 and Supplementary Table 1) of the core part, oxygen was absent, indicating complete sulfurisation, and atomic composition ratio was found to be close to W:S = 1:2. Furthermore, the x-ray diffractogram of assembled WS_2 NTs sample (Supplementary Fig. 3) shows that the all major peaks are indexed by the 2*H* WS_2, suggesting that the whole sample including the core and shell is likely 2*H* WS_2. However, in contrast to the nicely observed lamella structure of the shell in the TEM, the TEM image of the core is not very clear (Supplementary Fig. 1e and 1f). This indicates that the core structures are

highly disordered. Observation of the Little-Parks effect implies that only the outer shell region contributes to superconductivity, being consistent with the above observations.

Supplementary Fig. 4 shows the distribution of the outer diameter of the NTs. This analysis is based on about 50 NTs examined by TEM. The histogram shows a broad peak around 100 nm. This diameter is comparable to the values estimated from the Little-Parks oscillations.

Supplementary Fig. 5 displays the simulated electron diffraction patterns for three typical NTs of 2H WS_2 using a software Recipro ver.4.234 (http://pmsl.planet.sci.kobe-u.ac.jp/~seto/?lang=en).

The zig-zag type NT (Supplementary Fig. 5a) shows an upright hexagonal diffraction pattern (Supplementary Fig. 5d), while the arm-chair type NT (Supplementary Fig. 5c) is tilted by 30 degrees from the tube axis (Supplementary Fig. 5f). For the simulation of the chiral NTs, we take a tilted NT (Supplementary Fig. 5b) whose diffraction pattern is also tilted with the chiral angle θ ($0 < \theta < 30^o$). Because of the contribution from top and bottom surfaces of the tube, the diffraction patterns of the chiral nanotubes should be a double hexagons with tilted angles from the tube axis.

Supplementary Fig. 6-9 are typical diffraction images of WS_2 NTs. The diffraction pattern of tube A in Supplementary Fig. 6 are formed of one hexagon aligned to the tube axis (yellow line) and a set of two tilted hexagons (white lines), indicating that tube A is composed of the zig-zag and chiral nanotubes. On the other hand, tube B in Supplementary Fig. 7 are formed of one hexagon with tilting angle of 30° together with a pair of tilted hexagons, indicating that tube B involves the armchair and chiral nanotubes. Supplementary Fig. 8 and 9 show more complicated diffraction patterns, proving that tube C and D include tubes with a variety of chirality.

The observed electron diffraction patterns shown in Supplementary Fig. 6-9 imply that almost all the multi-walled WS_2 NTs contain chiral components.

In the main text, we show the colored Scanning Electron Microscope (SEM) image of WS_2 NT device in Fig.1e. The original image is shown in Supplementary Fig. 10.

Supplementary Note 2: Enhancement of the upper critical field

In this section, we argue on the enhancement of the upper critical field mentioned in the main text.

The Pauli limit is the upper critical field that breaks the spin-singlet Cooper pairs due to the Pauli paramagnetism in the presence of external magnetic field based on the weak coupling Bardeen–Cooper–Schrieffer (BCS) theory, which is also known as Chandrasechar-Clogston limit[1,2].

Phenomenologically speaking, the upper critical field H_P can be estimated when the Pauli paramagnetic energy $E_P = \frac{1}{2}\chi_P H_P^2$ is equal to the superconducting condensation energy $E_C = \frac{1}{2}N(E_F)\Delta^2$, where $\chi_P = \frac{1}{2}(g\mu_B)^2 N(E_F)$ is the spin susceptibility in normal state, Δ is the superconducting gap at zero temperature, $N(E_F)$ is the density of state at the Fermi level E_F in the normal state, g is the spectroscopic splitting factor of electrons ($g = 2$ for free electrons), and μ_B is the Bohr magneton. Thus the Pauli limit H_P is estimated to be $H_P = \frac{\sqrt{2}}{g\mu_B}\Delta$. According to the BCS theory, the superconducting gap Δ and superconducting critical temperature T_C obeys an universal value as $\Delta \approx 1.76k_B T_C$, where k_B denote the Boltzmann constant. Hence, the Pauli limit at zero temperature is estimated to be $H_P = \frac{\sqrt{2}}{g\mu_B}(1.76k_B T_C) \approx 1.84T_C$.

For superconducting WS_2 nanotubes, however, the observed upper critical field in the $H \parallel z$ configuration exceeds the Pauli paramagnetic limit discussed above, indicating the potential mechanism listed below which can explain the enhancement of the upper critical field.

In a superconductor with spin-orbit interaction, the enhancement of Pauli limit has been often discussed by spin-orbit scattering and spin-momentum (or spin-valley) locking.

(1) Enhanced Pauli limit of the two-dimensional (2D) superconductivity in transition metal dichalcogenides (TMDs) has been discussed very recently in terms of the Ising pairing protected by out-of-plane Zeeman-type spin-valley locking[3-5]. Since the multi-walled nanotube can be seen as the stacking of inequivalent curved 2D monolayers, the similar mechanism should be considered for the enhancement of Pauli limit in superconducting WS_2 nanotubes. We note that three-dimensional nature induced by the electrochemical doping cannot be also negligible, which can explain the smaller upper critical field in this system than pure 2D cases.

(2) In a dirty-limit superconductor, on the other hand, spin-orbit scattering mechanism is dominated[6], in which the spin paramagnetism is suppressed due to the random spin orientation by scattering, resulting in the decrease of Pauli spin split energy. Since the disorder is potentially caused during the intercalation process, the spin-orbit scattering effect may also contribute to the enhanced Pauli limit in superconducting WS_2 nanotubes.

In addition, the effect of the nontrivial Cooper pairing has been also discussed in the noncentrosymmetric and low dimensional superconductors.

(3) In a noncentrosymmetric superconductor, the symmetry allows the mixing of the spin singlet pairing and spin triplet pairing. Since the triplet pairing is robust against the external magnetic field, the mixing of spin singlet and spin triplet pairing cause the enhancement of the upper critical field, which should be taken into account in superconductivity of chiral WS_2 nanotubes.

(4) Another effect of the nontrivial pairing under magnetic field is known as the Fulde-Ferrell-Larkin-Ovchinnikov (FFLO) state[7-9]. In FFLO state, Cooper pairs have non-zero momentum, which cause the reduction of the Pauli spin split energy and resultant enhancement of the upper critical field. For one dimensional (1D) case, upper critical field even infinitely diverges at zero temperature[9]. Because of the superconductivity has been observed in low dimensional nanotube structure, such FFLO state may plays an important role for the enhancement of Pauli limit.

The detailed mechanism of the superconductivity and resultant Cooper pairing in a chiral WS_2 nanotube should be further pursued in the future.

Supplementary Note 3: Second harmonic signals in AC resistance

On the basis of symmetry arguments, the electric resistance in the case of parallel magnetic field can be expressed as

$$R(I,H) = R(-I,-H) = R_0(1 + \alpha I^2 + \beta(\mu_0 H)^2 + \gamma I \mu_0 H) \tag{1}$$

up to second order terms. Here, R_0, I and H represent the resistance at zero magnetic field, electric current and external magnetic field, respectively. For the non-chiral NTs with inversion symmetry, the last term in Supplementary Eq. 1 becomes zero, which is protected by the relation $R(I,H) = R(-I,H)$. However, if the tube is chiral, this term can be non-zero reflecting the broken inversion symmetry.

The current dependent term in Supplementary Eq. 1 leads to the nonlinear behavior in the voltage which can be detected as the second harmonic signal in lock-in measurements. If we apply AC input current ($I = I_0 \sin \omega t$), then the second harmonic signal due to the chiral term can be expressed as follows.

$$V^{2\omega}(t) = \gamma R_0 \mu_0 H I_0 \sin \omega t \cdot I_0 \sin \omega t = \gamma R_0 \mu_0 H I_0^2 \sin^2 \omega t$$

$$= \frac{1}{2}\gamma R_0 \mu_0 H I_0^2 \left\{1 + \sin\left(2\omega t - \frac{\pi}{2}\right)\right\} \tag{2}$$

According to Supplementary Eq. 2, there is a $\frac{\pi}{2}$ phase shift between the first and second harmonic signal. During the measurement, we picked up the y-component of the second harmonic signal reflecting the $\frac{\pi}{2}$ phase shift, and confirmed that the x-component (of the second harmonics) is almost zero.

In the main text, we show the raw data of $R^{2\omega}$, in which both symmetric and antisymmetric components with respect to the external magnetic field are included. Symmetric signals

possibly originate from the inhomogeneity of the device. On the other hand, antisymmetric components indicate nonreciprocal transport.

In Supplementary Fig. 12a, we calculate the antisymmetrized second harmonic components from the measured signals. There is a clear oscillating behavior in the low magnetic field region as discussed in the main text. In the broader components, on the other hand, there are several characteristic maxima or minima. The temperature dependence of each maximum and minimum position is roughly estimated and shown in Supplementary Fig. 12b, which shows the close correlation with the upper critical field. It seems that nonreciprocal signal from superconductivity with a specific chirality shows one peak structure around H_{c2}. Thus, the multi-peak structure possibly reflects the superposition of the signals from several superconducting layers with different chirality, T_c and H_{c2}, which need to be further studied in the future.

Supplementary References

[1] Chandrasekhar, B. S. A NOTE ON THE MAXIMUM CRITICAL FIELD OF HIGH-FIELD SUPERCONDUCTORS. *Appl. Phys. Lett.* **1**, 7-8 (1962)

[2] Clogston, A. M. UPPER LIMIT FOR THE CRITICAL FIELD IN HARD SUPERCONDUCTORS. *Phys. Rev. Lett.* **9**, 266-267 (1962)

[3] Saito, Y. *et al.* Superconductivity protected by spin-valley locking in ion-gated MoS_2. *Nature Phys.* **12**, 144-149 (2016)

[4] Lu, J. M. *et al.* Evidence for two-dimensional Ising superconductivity in gated MoS_2. *Science* **350**, 1353-1357 (2015)

[5] Xi, X. *et al.* Ising pairing in superconducting $NbSe_2$ atomic layers. *Nature Phys.* **12**, 139-143 (2016)

[6] Klemm, R. A., Luther, A., and Beasley, M. R. Theory of the upper critical field in layered superconductors. *Phys. Rev. B* **12**, 877-891 (1975)

[7] Fulde, P. and Ferrell, R. A. Superconductivity in a Strong Spin-Exchange Field. *Phys. Rev.* **135**, A550-A563 (1964)

[8] Larkin, A. I. and Ovchinnikov, Y. N. Nonuniform state of superconductors. *Sov. Phys. JETP* **20**, 762-769 (1965)

[9] Matsuda, Y. and Shimahara H. Fulde-Farrell-Larkin-Ovchinnilov State in Heavy Fermion Superconductors. *J. Phys. Soc. Jpn.* **76**, 051005 (2007)
